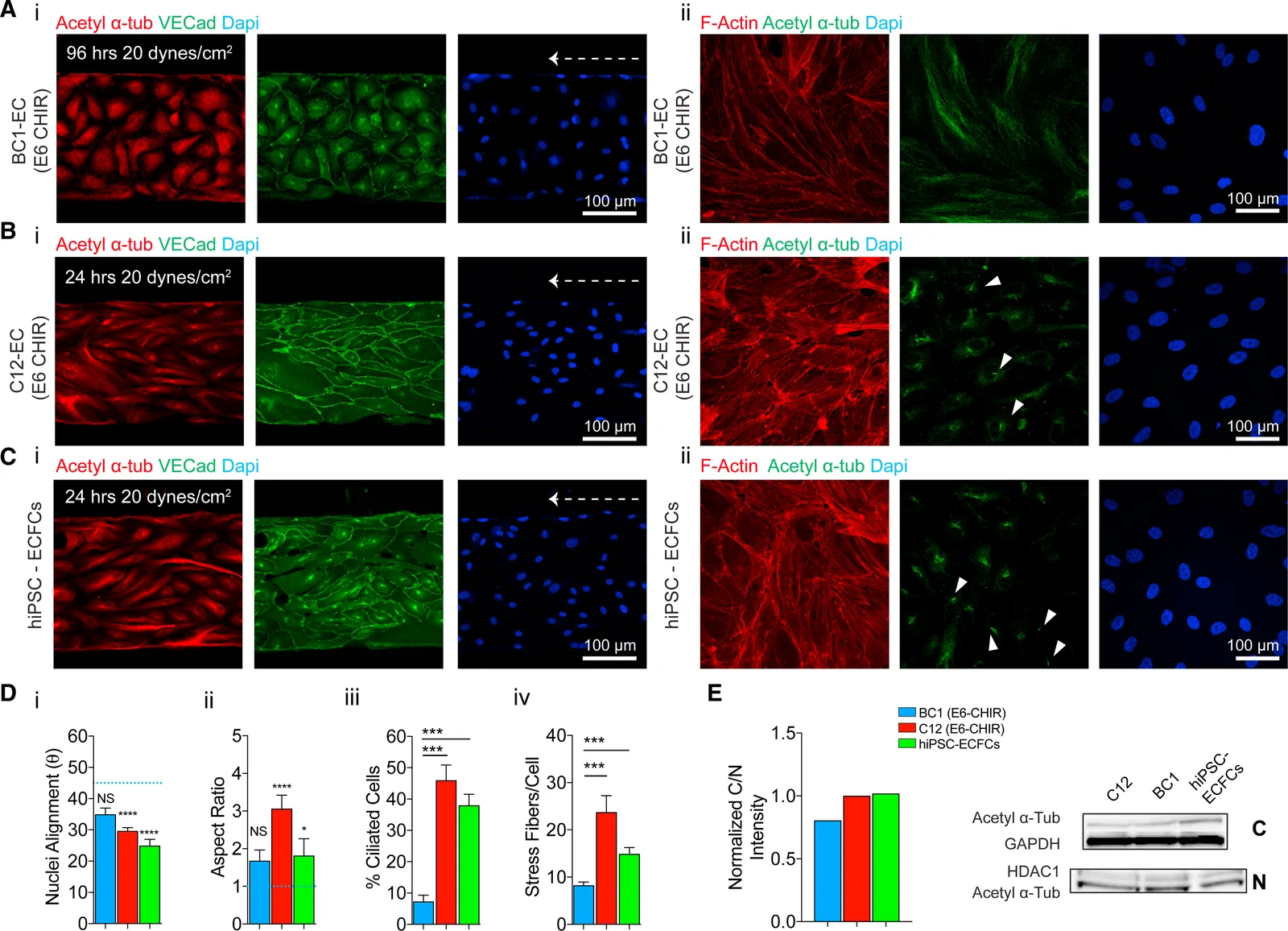# Differential HDAC6 Activity Modulates Ciliogenesis and Subsequent Mechanosensing of Endothelial Cells Derived from Pluripotent Stem Cells

**DOI:** 10.1016/j.celrep.2018.08.005

**Published:** 2018-08-14

**Authors:** Quinton Smith, Bria Macklin, Xin Yi Chan, Hannah Jones, Michelle Trempel, Mervin C Yoder, Sharon Gerecht

Due to a miscommunication during the proof stage, the Figure 3 included in this article as originally published online was an accidental duplication of Figure 2. Figure 3 has now been corrected online and is also shown below.

## Figures and Tables

**Figure:**